# Estimating the asymptomatic proportion of coronavirus disease 2019 (COVID-19) cases on board the Diamond Princess cruise ship, Yokohama, Japan, 2020

**DOI:** 10.2807/1560-7917.ES.2020.25.10.2000180

**Published:** 2020-03-12

**Authors:** Kenji Mizumoto, Katsushi Kagaya, Alexander Zarebski, Gerardo Chowell

**Affiliations:** 1 Graduate School of Advanced Integrated Studies in Human Survivability, Kyoto University Yoshida-Nakaadachi-cho, Sakyo-ku, Kyoto, Japan; 2 Hakubi Center for Advanced Research, Kyoto University, Yoshidahonmachi, Sakyo-ku, Kyoto, Japan; 3 Department of Population Health Sciences, School of Public Health, Georgia State University, Atlanta, Georgia, United States; 4 Seto Marine Biological Laboratory, Field Science, Education and Reseach Center, Kyoto University, Shirahama-cho, Nishimuro-gun, Wakayama, Japan; 5 Department of Zoology, University of Oxford, Oxford, United Kingdom

**Keywords:** corona, COVID-19, outbreak, Japan, asymptomatic, quarantine

## Abstract

On 5 February 2020, in Yokohama, Japan, a cruise ship hosting 3,711 people underwent a 2-week quarantine after a former passenger was found with COVID-19 post-disembarking. As at 20 February, 634 persons on board tested positive for the causative virus. We conducted statistical modelling to derive the delay-adjusted asymptomatic proportion of infections, along with the infections’ timeline. The estimated asymptomatic proportion was 17.9% (95% credible interval (CrI): 15.5–20.2%). Most infections occurred before the quarantine start.

An outbreak of coronavirus disease 2019 (COVID-19) unfolded on board a Princess Cruises’ ship called the Diamond Princess. Shortly after arriving in Yokohama, Japan, this ship had been placed under quarantine orders from 5 February 2020, after a former passenger had tested positive for the virus responsible for the disease (i.e. severe acute respiratory syndrome coronavirus 2; SARS-CoV-2), subsequent to disembarking in Hong Kong. In this study, we conducted a statistical modelling analysis to estimate the proportion of asymptomatic individuals among those who tested positive for SARS-CoV-2 on board the ship until 20 February 2020 included, along with their times of infections. The model accounted for the delay in symptom onset and also for right censoring, which can occur due to the time lag between a patient’s examination and sample collection and the development of illness.

## Epidemiological description and data

By 21 February 2020, 2 days after the scheduled 2-week quarantine came to an end, a total of 634 people including one quarantine officer, one nurse and one administrative officer tested positive for SARS-CoV-2. These individuals were among a total of 3,711 passengers and crew members on board the vessel.

Laboratory testing by PCR had been conducted, prioritising symptomatic or high-risk groups.

Daily time series of laboratory test results for SARS-CoV-2 (both positive and negative), including information on presence or absence of symptoms from 5 February 2020 to 20 February 2020 were extracted from secondary sources [1]. The reporting date, number of tests, number of persons testing positive by PCR (i.e. cases) and number of symptomatic and asymptomatic cases at the time of sample collection are provided, while the time of infection and true asymptomatic proportion are not available.

A total of 634 people tested positive among 3,063 tests as at 20 February 2020. Of 634 cases, a total of 313 cases were female and six were aged 0–19 years, 152 were aged 20–59 years and 476 were 60 years and older (Figure). Cases were from a total of 28 countries, with most being nationals of six countries, namely Japan (n = 270 cases), the United States (n = 88 cases), China (n = 58 cases; including 30 from Hong Kong), the Philippines (n = 54 cases), Canada (n = 51 cases) and Australia (n = 49 cases).

**Figure:**
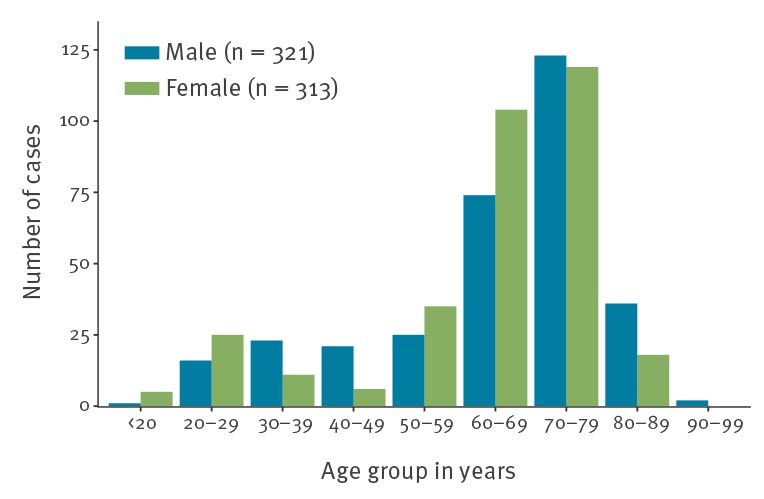
Age distribution of reported coronavirus disease 2019 cases on board the Diamond Princess cruise ship stratified by sex, Yokohama, Japan, 20 February 2020 (n = 634 cases)

Of the 634 confirmed cases, a total of 306 and 328 were reported to be symptomatic and asymptomatic, respectively. The proportion of asymptomatic individuals appears to be 16.1% (35/218) before 13 February, 25.6% (73/285) on 15 February, 31.2% (111/355) on 16 February, 39.9% (181/454) on 17 February, 45.4% (246/542) on 18 February, 50.6% (314/621) on 19 February and 50.5% (320/634) on 20 February (Table). Soon after identification of the first infections, both symptomatic and asymptomatic cases were transported to designated medical facilities specialised in infectious diseases in Japan. However, these patients were treated as external (imported) cases, and a detailed description of their clinical progression is not publicly available.

**Table:** Data on characteristics, test results for severe acute respiratory syndrome coronavirus 2, and disembarking of passengers and crew of the Diamond Princess cruise ship, Yokohama, Japan, February 2020 (n = 3,711)

Date (2020)^a^	Number of passengers and crew members on board	Number of disembarked passengers and crew members (cumulative)	Number of tests	Number of tests (cumulative)	Number of individuals testing positive	Number of individuals testing positive (cumulative)	Number of symptomatic cases	Number of asymptomatic cases	Number of asymptomatic cases (cumulative)
5 Feb	3,711	NA	31	31	10	10	NA	NA	NA
6 Feb	NA	NA	71	102	10	20	NA	NA	NA
7 Feb	NA	NA	171	273	41	61	NA	NA	NA
8 Feb	NA	NA	6	279	3	64	NA	NA	NA
9 Feb	NA	NA	57	336	6	70	NA	NA	NA
10 Feb	NA	NA	103	439	65	135	NA	NA	NA
11 Feb	NA	NA	NA	NA	NA	NA	NA	NA	NA
12 Feb	NA	NA	53	492	39	174	NA	NA	NA
13 Feb	NA	NA	221	713	44	218	NA	NA	NA
14 Feb	3,451	260^b^	NA	NA	NA	NA	NA	NA	NA
15 Feb	NA	NA	217	930	67	285	29	38	73^c^
16 Feb	NA	NA	289	1,219	70	355	32	38	111
17 Feb	3,183	528^b^	504	1,723	99	454	29	70	181
18 Feb	NA	NA	681	2,404	88	542	23	65	246
19 Feb	NA	NA	607	3,011	79	621	11	68	314
20 Feb	NA	NA	52	3,063	13	634	7	6	320

NA: data not available.

^a ^Reported date.

^b ^As this is a cumulative number, the exact date of disembarking is unavailable.

^c ^As this is a cumulative number, the reported date for 35 asymptomatic cases are unavailable.

The asymptomatic proportion was defined as the proportion of asymptomatically infected individuals among the total number of infected individuals.

## Statistical modelling

Here, we describe the statistical model that was employed to estimate the asymptomatic proportion using the time-series dataset described above.

The reported asymptomatic cases consists of both true asymptomatic infections and cases who had not yet developed symptoms at the time of data collection but became symptomatic later, i.e. the data are right censored. Each datum consists of an interval of time during which the individual may have been infected and a binary variable indicating whether they were symptomatic as at 18 February.

For individual *i* let [*a_i_*_,_ *b_i_*] denote the interval during which they may have been infected and *c* represents the censor date of observation of being symptomatic. The (unknown) time at which individual *i* was infected is denoted *X_i_* and, if they develop symptoms, let *D_i_* denote the delay from the time of infection until the time they are symptomatic, with cumulative density function (CDF), *F*_D_. The asymptomatic proportion, *p*, is the probability an individual will never develop symptoms.

Given an individual was exposed during the interval [*a_i_*_,_ *b_i_*] the probability for them being asymptomatic at time *c* is

gx,p=p+(1-p)(1-FDc-x) if they do not have symptoms,FDc-x             if they do have symptoms,

Given they were infected at time x for some *a* ≤ *x* ≤ *b*. Since the natural history of each individual’s infection is independent, the likelihood function is just the product of the *g*(*X*_i_, *p*) for each individual. Previous work on COVID-19 suggests that the distribution of the delay, *D*, between infection and onset of symptomatic infection (i.e. the incubation period) follows a Weibull distribution, with a mean and standard deviation at 6.4 and 2.3 days [2].

The observations were treated as survival data with right censoring. The probability of being asymptomatic given infection along with the infection time of each individual were estimated in a Bayesian framework using Hamiltonian Monte Carlo (HMC) algorithm. A detailed description of the model used and the computation is provided in the Supplementary material.

## Findings from the real-time outbreak analysis

The posterior median estimate of the true proportion of asymptomatic individuals among the reported asymptomatic cases is 0.35 (95% credible interval (CrI): 0.30–0.39), with the estimated total number of the true asymptomatic cases at 113.3 (95%CrI: 98.2–128.3) and the estimated asymptomatic proportion (among all infected cases) at 17.9% (95%CrI: 15.5–20.2%).

We conducted sensitivity analyses to examine how varying the mean incubation period between 5.5 and 9.5 days affects our estimates of the true asymptomatic proportion. Estimates of the true proportion of asymptomatic individuals among the reported asymptomatic cases are somewhat sensitive to changes in the mean incubation period, ranging from 0.28 (95%CrI: 0.23–0.33) to 0.40 (95%CrI: 0.36–0.44), while the estimated total number of true asymptomatic cases range from 91.9 (95%CrI: 75.2–108.7) to 130.8 (95%CrI: 117.1–144.5) and the estimated asymptomatic proportion ranges from 20.6% (95%CrI: 18.5–22.8%) to 39.9% (95%CrI: 35.7–44.1%).

Heat maps were used to display the density distribution of infection timing by individuals (Supplementary Figures S1 and S2) where the vertical line corresponds to the date of 5 February 2020. Among the symptomatic cases, the infection timing appears to have occurred just before or around the start of the quarantine period (Supplementary Figure S1), while the infection timing for asymptomatic cases appears to have occurred well before the start of the quarantine period (Supplementary Figure S2).

## Discussion

Since COVID-19 emerged in the city of Wuhan, China, in December 2019, thousands of people have died from SARS-CoV-2, especially in the Province of Hubei. Meanwhile hundreds of imported and resulting secondary cases have been reported in multiple countries as at 29 February 2020 [3].

The clinical and epidemiological characteristics of COVID-19 continue to be investigated as the virus further transmits through the human population [2,4]. While reliable estimates of the reproduction number and the death risk associated with COVID-19 are crucially needed to guide public health policy, another key epidemiological parameter that could inform the intensity and range of social distancing strategies to combat COVID-19 is the asymptomatic proportion, which is broadly defined as the proportion of asymptomatic infections among all the infections of the disease. Indeed, the asymptomatic proportion is a useful quantity to gauge the true burden of the disease and better interpret estimates of the transmission potential. This proportion varies widely across infectious diseases, ranging from 8% for measles and 32% for norovirus infections up to 90–95% for polio [5-7]. Most importantly, for measles and norovirus infections, it is well established that asymptomatic individuals are frequently able to transmit the virus to others [8,9]. Currently, there is no clear evidence that COVID-19 asymptomatic persons can transmit SARS-CoV-2, but there is accumulating evidence indicating that a substantial fraction of SARS-CoV-2 infected individuals are asymptomatic [10-12].

As an epidemic progresses over time, suspected cases are examined and tested for the infection using laboratory diagnostic methods. Then, time-stamped counts of the test results stratified according to the presence or absence of symptoms at the time of testing are often reported in near real-time. Nevertheless, it is important to note that the estimation of the asymptomatic proportion needs to be handled carefully since real-time outbreak data are influenced by the phenomenon of right censoring.

In this study, we conducted statistical modelling analyses on publicly available data to elucidate the asymptomatic proportion, along with the time of infection among the COVID-19 cases on board the Diamond Princess cruise ship.

Our estimated asymptomatic proportion is at 17.9% (95%CrI: 15.5–20.2%), which overlaps with a recently derived estimate of 33.3% (95% confidence interval: 8.3–58.3%) from data of Japanese citizens evacuated from Wuhan [13]. Considering the reported similarity in viral loads between asymptomatic and symptomatic patients [14] and that transmission of SARS-CoV-2 by asymptomatic or paucisymptomatic cases may be possible, even though there is no clear evidence as yet of asymptomatic transmission, the relatively high proportion of asymptomatic infections could have public health implications. For instance, the United States Centers for Disease Control and Prevention recommends that contacts of asymptomatic cases self isolate for 14 days [15].

Most of the infections on board the Diamond Princess cruise ship appear to have occurred before or around the start of the 2-week quarantine that started on 5 February 2020, which further highlights the potent transmissibility of the SARS-CoV-2 virus, especially in confined settings. To further mitigate transmission of COVID-19 and bring the epidemic under control in areas with active transmission, it may be necessary to minimise the number of gatherings in confined settings.

Our study is not free from limitations. First, laboratory tests by PCR were conducted focusing on symptomatic cases especially at the early phase of the quarantine. If asymptomatic cases were missed as a result of this, it would mean we have underestimated the asymptomatic proportion. Second, it is worth noting that the passengers and crew whose data were employed in our analysis do not constitute a random sample from the general population. Considering that most of the passengers were 60 years and older, the nature of the age distribution may lead to underestimation if older individuals tend to experience more symptoms. An age standardised asymptomatic proportion would be more appropriate in that case. Third, the presence of symptoms in cases with COVID-19 may correlate with other factors unrelated to age including prior health conditions such as cardiovascular disease, diabetes, and/or immunosuppression. Therefore, more detailed data documenting the baseline health of the individuals including the presence of underlying diseases or comorbidities would be useful to remove the bias in estimates of the asymptomatic proportion.

In summary, we have estimated the proportion of asymptomatic cases among individuals who tested positive for SARS-CoV-2 along with the times of infection of confirmed cases on board the Diamond Princess cruise ship after adjusting for the delay in symptom onset and right censoring of the observations. 

## Supplementary Data

345000 Supplementary Material
