# Author’s correction for Euro Surveill. 2020;25(10)

**DOI:** 10.2807/1560-7917.ES.2020.25.22.20200604c

**Published:** 2020-06-04

**Authors:** Eurosurveillance editorial team

**Affiliations:** 1 European Centre for Disease Prevention and Control (ECDC), Stockholm, Sweden

In the article ‘Estimating the asymptomatic proportion of coronavirus disease 2019 (COVID-19) cases on board the Diamond Princess cruise ship, Yokohama, Japan, 2020’ by Mizumoto et al. published on 12 March 2020, a mistake in the equation presented in the article was corrected at the request of the authors. The correction only concerned the display of the equation, as calculations in the article had been carried out using the correct equation. The change was made on 3 June 2020.

