# Supplementary material for: Estimating the asymptomatic proportion of coronavirus disease 2019 (COVID-19) cases on board the Diamond Princess cruise ship, Yokohama, Japan, 2020
Source: Euro Surveill. 2020 Mar 12;25(10):2000180. doi: 10.2807/1560-7917.ES.2020.25.10.2000180 (PMC7078829; doi:10.2807/1560-7917.ES.2020.25.10.2000180)
Supplement: Supplementary Material [file 2000180_MIZUMOTO_Supplementary_Material.pdf]

Supplement S1:

Estimating the asymptomatic proportion of coronavirus disease 2019 (COVID-19) cases on board the Diamond Princess cruise ship, Yokohama, Japan, 2020

Mizumoto K, Kagaya K, Zarebski A, Chowell G.

Disclaimer

This supplementary material is hosted by *Eurosurveillance* as supporting information alongside the article 'Estimating the asymptomatic proportion of coronavirus disease 2019 (COVID-19) cases on board the Diamond Princess cruise ship, Yokohama, Japan, 2020', on behalf of the authors, who remain responsible for the accuracy and appropriateness of the content. The same standards for ethics, copyright, attributions and permissions as for the article apply. Supplements are not edited by *Eurosurveillance* and the journal is not responsible for the maintenance of any links or email addresses provided therein.

1. Supplementary
2. Appendix Figures S1, S2

Statistical Analysis

1. Bayesian Analysis

We are interested in estimating both the asymptomatic proportion, p, and the time of infection for each of the confirmed cases, i.e. the X_i. The prior distribution on X_i was uniform over $[a_i, b_i]$. We estimated model parameters using Hamiltonian Monte Carlo (HMC) with the No-U-Turn-Sampler (NUTS) provided by Stan accessed via the rstan interface. Point estimates and the corresponding 95% credibility intervals obtained from the posterior samples. All statistical analyses were conducted in R version 3.6.1 (R Foundation for Statistical Computing, Vienna, Austria).

2 Missing Data and Sensitivity Analysis

As for the 35 asymptomatic cases of the 73 cumulative asymptomatic cases reported on February 15, 2020, the date of reporting is not available. Considering the number of people that tested positive for each date, we randomly allocated the 35 asymptomatic cases from February 5 to February 13, in order to obtain the number of symptomatic and asymptomatic cases for each date before estimating the parameters. Moreover, we repeat this experiment 200 times, allowing us to take percentile points for the calculation of uncertainty bounds.

For sensitivity analyses, we examined the effect of varying the mean of the incubation period on p, using values for the mean incubation period in the range: 4.4 to 8.4 days.

3 Convergence Diagnostics and Quality Control

Posterior distributions of the model parameters were estimated based on sampling from the three Markov chains. For each chain, we drew 100,000 samples from the posterior distribution after a burn–in of 20,000 iterations. Convergence of MCMC chains were

evaluated using the potential scale reduction statistic [1]. Estimates and 95% credibility intervals for these estimates are based on the posterior probability distribution of each parameter and based on the samples drawn from the posterior distributions.

Figure S1. Heat maps of the density distribution of infection timing by individuals, Symptomatic cases (N= 306)

Vertical axis represents each individual from 1 to N. Cases disembarked after testing positive for the disease. Day 1 corresponds to January 20, 2020, when the first symptomatic case embarked. The vertical line corresponds to February 5, 2020 when the quarantine period started.

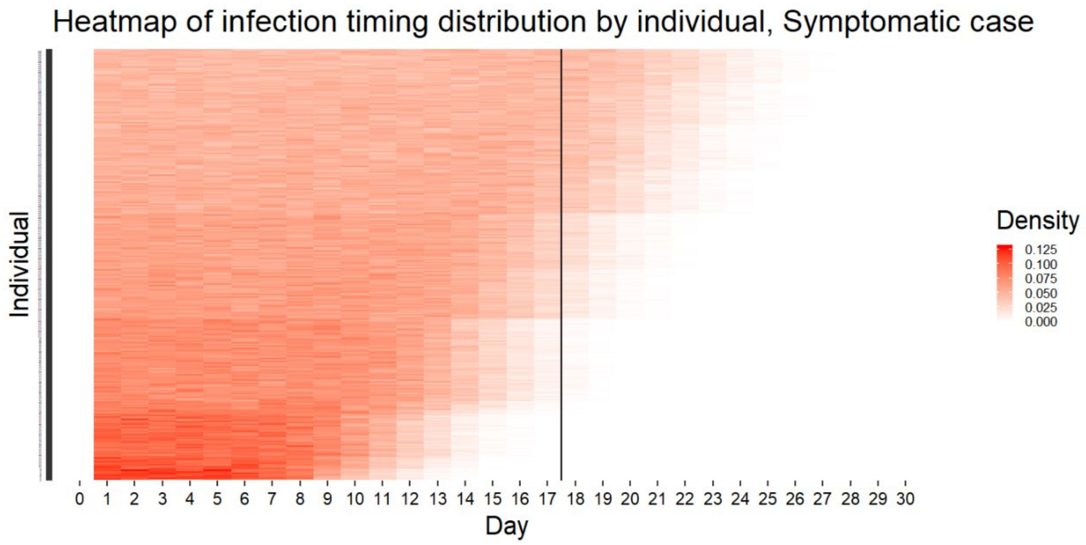

Figure S2. Heat maps of the density distribution of infection timing by individuals, Asymptomatic cases (N= 320)

Vertical axis represents each individual from 1 to N. Cases disembarked after testing positive for the disease. Day 1 corresponds to January 20, 2020, when the first symptomatic case embarked. The vertical line corresponds to February 5, 2020 when the quarantine period started.

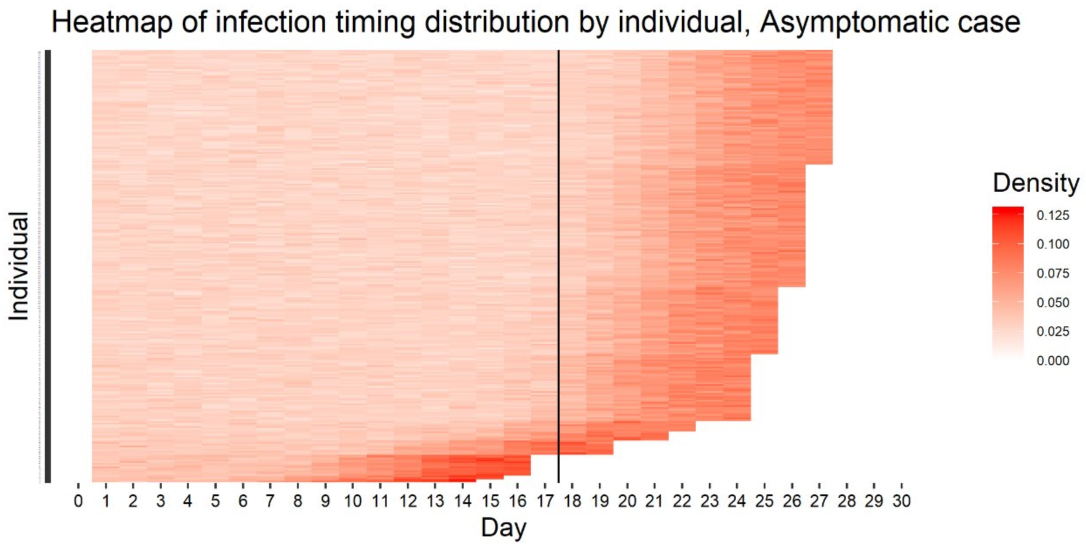

REFERENCES

1. Gamerman, D. & Lopes, H. F. Markov Chain Monte Carlo: Stochastic Simulation for Bayesian Interference. 2nd edn (Chapman & Hall/CRC, 2006).
